# The influence of immune regulation mediated by intestinal microbiota on postmenopausal osteoporosis and intervention strategies

**DOI:** 10.3389/fendo.2025.1720484

**Published:** 2026-01-05

**Authors:** Lili Wang, Shiqing Chen, Xiaoyu Cai, Yongquan Zheng, Caihong Zheng, Yao Yao

**Affiliations:** 1 Department of Pharmacy, Women’s Hospital School of Medicine Zhejiang University, Hangzhou, Zhejiang, China; 2 Department of Pharmacy, Affiliated Hangzhou First People’s Hospital, School of Medicine, Westlake University, Hangzhou, Zhejiang, China

**Keywords:** bone metabolism, gut microbiota, immune regulation, intervention strategies, PMO

## Abstract

Postmenopausal osteoporosis (PMO) is a common metabolic bone disease characterized by reduced bone mass and deteriorated bone microarchitecture, leading to an increased risk of fractures. In recent years, growing evidence has highlighted the role of gut microbiota and its immune-mediated regulation in the pathogenesis and progression of PMO. The gut microbiota modulates host immune responses, influencing the balance between bone resorption and bone formation. Estrogen deficiency after menopause disrupts gut microbiota composition, induces systemic inflammation, and promotes osteoclast activation, accelerating bone loss. Moreover, specific microbial communities and their metabolites, such as short-chain fatty acids (SCFAs), regulate bone metabolism by modulating immune cells, including T cells, B cells, and macrophages. Various microbiota-targeted interventions, such as probiotics, prebiotics, and fecal microbiota transplantation (FMT), have shown potential in improving bone health. However, several challenges remain, including individual variability in microbiota composition, the long-term effects of interventions, and their clinical applicability. Further investigations into the gut microbiota-mediated immune regulation of PMO may provide novel insights and therapeutic strategies for osteoporosis prevention and treatment.

## 1 Introduction

Postmenopausal osteoporosis (PMO) is an estrogen deficiency-mediated skeletal disorder characterized by reduced bone mineral density (BMD) and microarchitectural deterioration of bone tissue, leading to increased bone fragility and consequently heightened fracture susceptibility in postmenopausal women (1). Epidemiological studies demonstrate that osteoporosis prevalence exceeds 30% in Chinese women aged 50 years and older, with site-specific rates of 37% at the lumbar spine and 16% at the total hip (2, 3). Beyond elevating fracture risks (e.g., hip and vertebral fractures), PMO severely compromises mobility, induces chronic pain, and diminishes quality of life, incurring substantial socioeconomic burdens (4). Annual osteoporotic fracture incidence is projected to surge by 135% (from 6.9 million to 16.2 million cases) and associated economic costs by 121% (from $29.9 billion to $65.9 billion USD) during the 2020–2040 period (5).

The gut microbiota is an extremely complex and dynamically changing microbial ecosystem, primarily composed of bacteria, while also encompassing archaea, fungi, viruses, and protozoa (6). The human gut microbiota exhibits a unique spatial distribution pattern, with microbial density and diversity showing a sharp increasing trend along the gastrointestinal tract: the stomach and proximal small intestine are nearly sterile (≤10³–10^4^ CFU/mL), while the colon reaches high density (10¹¹–10¹² CFU/g contents) and high complexity (7). The core of this microbiota is predominantly dominated by four bacterial phyla: *Firmicutes*, *Bacteroidetes*, *Actinobacteria*, and *Proteobacteria* (8). The gut microbiota can ferment indigestible complex carbohydrates such as dietary fiber in the human body to produce SCFAs (e.g., butyric acid, acetic acid, propionic acid), which in turn participate in systemic energy metabolism and blood glucose regulation. Meanwhile, they can also synthesize vitamin K and B vitamins (e.g., vitamin B12, folic acid) to assist the host in nutrient absorption, and influence fat digestion and cholesterol metabolism by converting bile acids (BAs) (9).As a “barrier guardian,” the microbiota competitively inhibits pathogen adhesion through colonization resistance and secretes antimicrobial substances. It simultaneously stimulates mucus secretion, maintains an acidic environment, and promotes intestinal epithelial tight junction protein expression—collectively building robust physical, chemical, and biological barriers that prevent harmful substances from leaking into the bloodstream (“leaky gut”) (10). It promotes regulatory T-cell differentiation to maintain immune tolerance, prevents excessive inflammatory responses, continuously activates immune cells, enhances antimicrobial peptide secretion, and balances gut and systemic immune defenses.

Based on the above content, we further propose the theoretical model of the “estrogen-gut microbiota-immunity-bone axis”. In animal experiments, ovariectomized (OVX) female rats simulating estrogen deficiency show significant changes in gut microbiota structure: a decrease in the number of beneficial bacteria, an increase in harmful bacteria, accompanied by immune system activation and rapid bone loss (11). Notably, estrogen replacement therapy (ERT) can restore the gut microbiota to a state close to normal, inhibit excessive immune activation, and significantly alleviate bone loss (12). Similarly, in mouse models, sufficient estrogen maintains the ability of gut microbiota to metabolize SCFAs; these SCFAs maintain the homeostatic balance of bone remodeling by regulating immune cell activity and inhibiting the production of pro-inflammatory cytokines. Conversely, estrogen deficiency impairs the ability of microbiota to produce SCFAs, leading to abnormal activation of immune cells, massive release of inflammatory factors, enhanced bone resorption and weakened bone formation. Clinical observations have further verified this model: postmenopausal women, due to the decline of ovarian function and the sharp drop in estrogen levels, have significantly reduced diversity and abundance of gut microbiota, and are prone to immune dysfunction and osteoporosis (13).

Our previous research focused on the mechanism of action of gut microbiota and their metabolites in the immune response of rheumatoid arthritis (RA) (14) as well as the mechanism of PMO-related bone loss (15). Based on these research findings, we further conducted a systematic review of the microbiota-mediated immunomodulatory mechanisms in PMO, and comprehensively evaluated the translational application potential of microbiota modulation strategies in this field.

## 2 The mechanism of crosstalk between the gut microbiota and the immune system

### 2.1 The gut microbiota in shaping and regulating the immune system

As a complex microecological community in the human intestinal tract, the gut microbiota not only participates deeply in the basic physiological processes of the body, but also plays a crucial role in the development and maturation, functional maintenance, and homeostatic balance of the immune system. Its regulation of the immune system is continuous, spanning the entire life cycle of the host from the embryonic stage to adulthood. At the molecular level, the gut microbiota can activate immune cells through interaction with pattern recognition receptors (PRRs), thereby maintaining the homeostatic balance of the intestinal microecology—a process that forms an important defensive barrier for the host to resist the invasion of pathogenic microorganisms and protect its own health.

#### 2.1.1 Immunomodulatory effects of gut microbiota across different developmental stages of the host

During the embryonic stage, although the traditional view holds that the fetus is in a sterile environment, recent studies have confirmed that maternal gut microbiota can influence the initial programming of the fetal immune system through metabolite-mediated transplacental signals. During this process, short-chain fatty acids and tryptophan metabolites (e.g., indole-3-lactic acid) produced by the maternal microbiota collectively regulate the epigenetic modification of fetal immune-related genes through specific mechanisms: among short-chain fatty acids, butyrate exerts its effect mainly by directly inhibiting histone deacetylases (HDACs), while propionate and acetate take the activation of G protein-coupled receptor 43 (GPR43) and subtypes such as GPR41 as the core pathway, accompanied by a mild auxiliary effect of inhibiting HDACs; tryptophan metabolites participate in the regulation by activating GPR43 or inhibiting HDACs. These metabolites synergistically act on key immune genes including forkhead box P3 (Foxp3) and interleukin 10 (IL-10), ultimately forming the “innate imprint” of immune tolerance (16, 17).

The neonatal period is a critical window for gut microbiota colonization and the rapid development of the immune system (18). Delivery methods and feeding patterns directly affect the initial colonization characteristics of the microbiota: neonates delivered vaginally preferentially acquire maternal vaginal microbiota (e.g., *Lactobacillus*), while those delivered by cesarean section are more likely to be colonized by Staphylococcus from the environment. Breastfeeding selectively enriches microbiota such as *Bifidobacterium* and *Lactobacillus* through human milk oligosaccharides (HMOs) (19). The exopolysaccharides (EPS) secreted by these colonizing microorganisms—such as those produced by lactic acid bacteria—possess significant health-promoting properties: they can stimulate the proliferation of beneficial bacteria, inhibit the adhesion of harmful bacteria to the intestinal epithelium, enhance the integrity of the intestinal barrier by upregulating the expression of tight junction proteins, and regulate immune system function through direct or indirect interactions with Toll-like receptors (20). Another study has confirmed that EPS produced by Lactobacillus and Bifidobacterium can effectively attenuate the inflammatory response induced by enterotoxigenic Escherichia coli in porcine intestinal epithelial cells (21). In addition, numerous studies have reported the regulatory effects of EPS on specific immune cell types and immune responses. Polysaccharide A (PSA) is one of the eight polysaccharides produced by Bifidobacterium fragilis, and its regulatory effects on dendritic cells (DCs) and T cell responses have been elucidated in multiple studies. Bifidobacterium fragilis can secrete outer membrane vesicles containing PSA; these vesicles can be taken up by DCs and induce the development of plasmacytoid DCs, which produce IL-12, TNF-α, and IFN-γ in a TLR2-dependent manner, thereby regulating the cytokine environment in the body (22, 23). In summary, exopolysaccharides may maintain the balance between immune tolerance and pro-inflammatory responses through the precise regulation of immune cells and cytokine networks, thus constituting the core mechanism for neonates to establish immune tolerance (24).

#### 2.1.2 Pathways of gut microbiota-mediated immune cell activation via PRRs

The dynamic interaction between the gut microbiota and the host immune system is crucial for maintaining intestinal homeostasis and systemic immune balance, with its key molecular basis lying in the precise sensing and transduction of microbiota-derived signals by PRRs. As the host’s “molecular sentinels”, PRRs can specifically recognize microbiota-derived pathogen-associated molecular patterns (PAMPs) or damage-associated molecular patterns (DAMPs) induced by the microbiota, and activate immune cells by initiating cascading signaling pathways (25).

**Figure 1:**
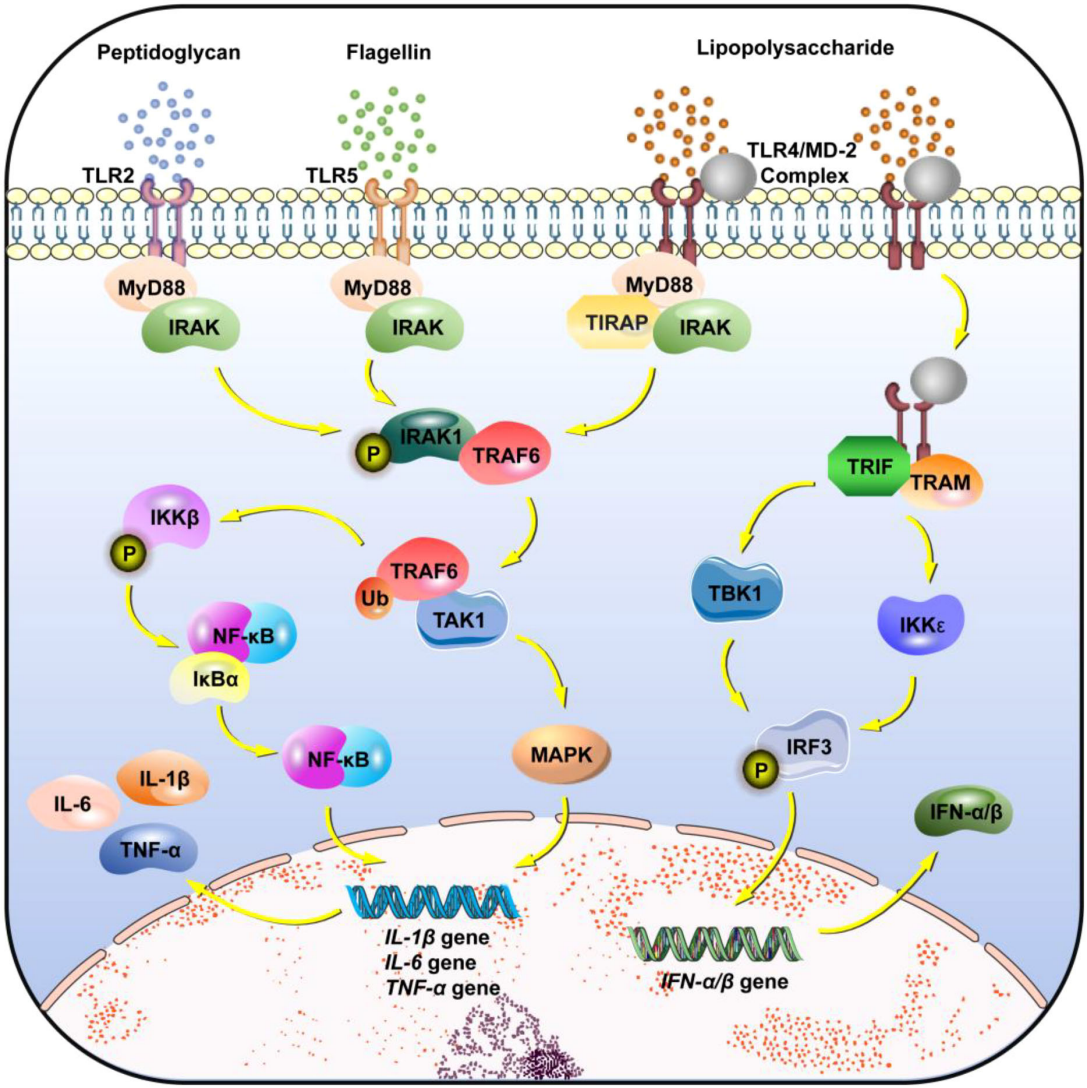
TLRs. (1) TLR2: Peptidoglycan activates TLR2, which binds MyD88 and recruits IRAK to form a “TLR2-MyD88-IRAK” complex. Activated IRAK activates TRAF6, which in turn activates TAK1. TAK1 phosphorylates IKKβ, leading to the degradation of IκBα and the release of NF-κB. NF-κB enters the nucleus to initiate the transcription of proinflammatory cytokine genes, such as IL-1β, IL-6, and TNF-α. TAK1 also activates the MAPK pathway to synergize with NF-κB in gene regulation. (2) TLR5: Flagellin activates TLR5, which recruits MyD88 and IRAK to form a signaling complex. Phosphorylated IRAK1 binds to and activates TRAF6 via ubiquitination, following the same mechanism as the TLR2 pathway. (3) TLR4: TLR4 forms a complex with MD-2 to recognize LPS and activates both the MyD88-dependent pathway and TRIF-dependent pathway. The MyD88-dependent pathway is the same as that of TLR2 and TLR5. For the TRIF-dependent pathway, LPS-induced endocytosis of the TLR4/MD-2 complex allows TLR4 to bind TRIF and recruit TRAM. TRIF activates TBK1 and IKKϵ, which phosphorylate IRF3. Phosphorylated IRF3 enters the nucleus to initiate the transcription of type I interferons.

Toll-like receptors (TLRs), the core membrane-bound pathway mediating the recognition of extracellular signals from the microbiota, are widely distributed on the surface of intestinal immune cells or endosomal membranes, with distinct subtypes exhibiting clear functional specialization in response to microbial signals (26) (**Figure 1**). TLR2, expressed on intestinal lamina propria macrophages and DCs, forms heterodimers with TLR1/6 to recognize peptidoglycan from Gram-positive bacteria. This interaction recruits myeloid differentiation primary response 88 (MyD88), activating nuclear factor-κB (NF-κB) and mitogen-activated protein kinases (MAPKs) signaling pathways. Consequently, proinflammatory cytokines (e.g., tumor necrosis factor-α (TNF-α), interleukin-6 (IL-6), and interleukin-1β (IL-1β) are secreted, enabling broad-spectrum immune defense against the microbiota. Toll-like receptor 4 (TLR4), expressed on intestinal macrophages, DCs, and vascular endothelial cells, requires complex formation with myeloid differentiation factor 2 (MD-2) to recognize lipopolysaccharide (LPS) from Gram-negative bacteria. This process triggers a dual signaling cascade. First, TLR4 forms a complex with MD-2 to recognize LPS, thereby activating both MyD88-dependent and the TIR-domain-containing adapter-inducing interferon (TRIF)-dependent pathways. Second, for the TRIF-dependent pathway, LPS-induced endocytosis of the TLR4/MD-2 complex allows TLR4 to bind TRIF and recruit TRIF-related adapter molecule (TRAM). TRIF then activates TANK-binding kinase 1 (TBK1) and inhibitor of nuclear factor-κB kinase ϵ (IKKϵ), which phosphorylate interferon regulatory factor 3 (IRF3). Phosphorylated IRF3 translocates into the nucleus and initiates the transcription of type I interferons. The MyD88-dependent pathway rapidly induces the secretion of proinflammatory factors to recruit neutrophils for clearance of invading microbiota, while the TIR-domain-containing adapter-inducing interferon (TRIF)-dependent pathway induces the secretion of type I interferons, promotes the maturation of DCs and differentiation of T cells, and balances inflammation and immune tolerance. DCs within Peyer’s patches highly express TLR5, which specifically recognizes flagellin from motile bacteria. Through MyD88-mediated signaling, TLR5 induces interleukin-18 (IL-18) secretion, promoting T cell differentiation into T helper 1 (Th1) cells and enhancing the clearance capacity of intracellular pathogens.

**Figure 2:**
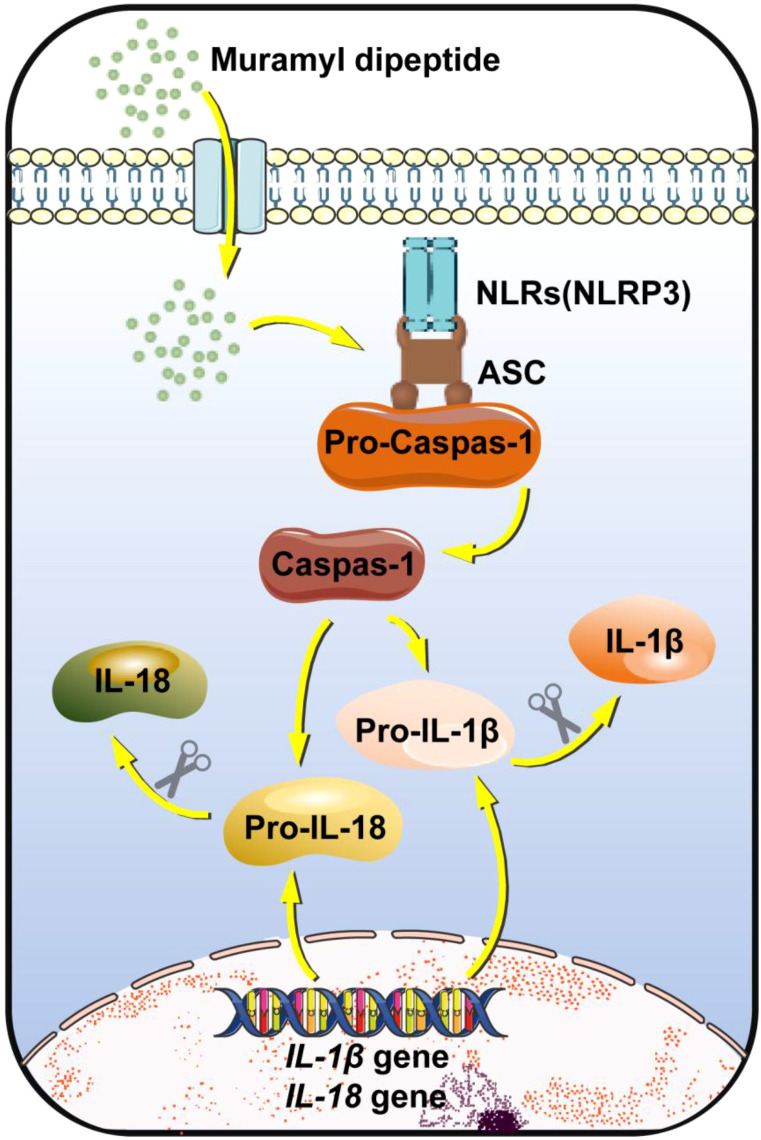
NLRs. NLRs are activated upon the entry of muramyl dipeptide into the cell cytoplasm. The core function of activated NLRs is to activate Caspase-1 via “inflammasomes” (e.g., the NLRP3 inflammasome), which in turn cleaves pro-IL-1β and pro-IL-18, ultimately promoting the maturation and release of IL-1β and IL-18.

The NOD-like receptor family pyrin domain containing 3 (NLRP3) inflammasome, a member of the nucleotide-binding oligomerization domain-like receptors (NLRs) family, is a proinflammatory pathway that mediates the recognition of intracellular signals from microbiota (27) (**Figure 2**). NLRP3 is localized in the cytoplasm of intestinal macrophages, neutrophils, and DCs. It activates the NF-κB pathway in immune cells by recognizing LPS, thereby inducing the gene expression of pro-interleukin-1β (pro-IL-1β) and pro-interleukin-18 (pro-IL-18). Meanwhile, NLRP3 recruits the adaptor protein apoptosis-associated speck-like protein containing a CARD (ASC), which then binds to pro-caspase-1 to assemble into mature inflammasomes. Active caspase-1 cleaves pro-IL-1β and pro-IL-18 into mature cytokines, which recruit more immune cells to accumulate and clear pathogenic bacteria.

The immunoregulatory network established by gut microbiota through TLRs and NLRs pathways represents the core mechanism underlying host adaptation to the intestinal microbial environment and maintenance of intestinal homeostasis. Through synergistic interaction, these two pathways form a “network loop”, which precisely regulates immune cell function and intestinal barrier function, and prevents excessive or insufficient immune responses.

### 2.2 Gut microbiota regulates immune cells and antibody production

The gut microbiota exerts a crucial regulatory role in B cells and their antibody production, with specific mechanisms involving multiple detailed aspects: Exposure to the gut microbiota not only promotes the continuous diversification of the B cell repertoire but also induces antibody production through two pathways, namely T cell-dependent and T cell-independent. Among these antibodies, IgA— the major secretory antibody at mucosal surfaces—serves as the core product and plays a key role in maintaining intestinal homeostasis (28). Although B cells are present in gut-associated lymphoid tissues such as Peyer’s patches and mesenteric lymph nodes prior to birth, antigens from the gut microbiota and metabolites like short-chain fatty acids strongly promote the differentiation of B cells into plasma cells in both mucosal and systemic sites. Additionally, the antibody production of T cell-dependent B cells is directly associated with exposure to microbial antigens (29). As key microbial metabolites, short-chain fatty acids can also effectively enhance the cellular metabolism of B cells. By supporting glycolysis and mitochondrial energy production, they provide the necessary energy and structural substances for B cell activation, differentiation, and antibody synthesis, ultimately optimizing the antibody defense function of the adaptive immune system and helping the host resist microbial invasion (30).

### 2.3 The reverse regulatory role of the immune system in the gut microbiota

There exists a complex and sophisticated bidirectional regulatory relationship between the intestinal microbiota and the host’s immune system. On one hand, symbiotic microbiota can initiate the synergistic effect of intestinal innate and adaptive immunity, thereby protecting the host from invasion by foreign pathogens and maintaining intestinal homeostasis (31). On the other hand, the maintenance of intestinal microbiota homeostasis also relies on the precise regulation of the host’s immune system. This “counteraction” is not a simple clearance effect, but rather achieves selective recognition, targeted regulation, and maintenance of dynamic balance of the microbiota through the coordination of immune cells and immune molecules.

#### 2.3.1 Mechanisms of gut microbiota selection, clearance, and homeostasis maintenance by immune cells and molecules

DCs, as critical bridges linking innate and adaptive immunity, play an irreplaceable role in maintaining gut microbiota homeostasis through precise sensing of microbial signals, regulation of immune response balance, and preservation of intestinal barrier integrity (32). Firstly, as “sensors” of microbial signals, DCs achieve selective recognition of commensals and pathogens via differential activation of PRRs: they downregulate proinflammatory PRRs (e.g., TLR4) or utilize C-type lectin receptors (e.g., DC-specific intercellular adhesion molecule-3-grabbing non-integrin) to avoid excessive responses to commensals, while efficiently recognizing pathogenic PAMPs (e.g., flagellin) through TLR5 or Nucleotide-binding oligomerization domain 2 (NOD2) (33). Secondly, as “protectors” of commensals, DCs induce immune tolerance by secreting transforming growth factor-β (TGF-β), retinoic acid, and IL-10 to promote regulatory T cells (Tregs) differentiation, which in turn inhibits proinflammatory effector T cells (Th1/Th17) via IL-10 and TGF-β (34). Thirdly, as “eliminators” of pathogens, CD103^+^CD11b^+^ DCs can respond to the stimulation of bacterial flagellin by expressing TLR5. They rapidly produce interleukin-23 (IL-23) to activate antibacterial inflammatory responses, and further drive the interleukin-22 (IL-22)-dependent production of regenerating islet-derived protein III gamma (RegIIIγ), thereby ultimately enhancing the defensive capacity of the intestinal mucosa against bacterial pathogens (35). In addition, DCs can also regulate the activity of a variety of immune cells within the intestine, including monocytes, macrophages, T cells, and B cells (36).

Intestinal macrophages serve as the first line of leukocyte defense against pathogen invasion (37). They not only block pathogen intrusion but also maintain intestinal homeostasis. Macrophages act as a key component of the human innate immune system by rapidly recognizing PAMPs on the bacterial surface (38, 39). When stimulated by bacterial LPS and the cytokine interferon-γ (IFN-γ), proinflammatory macrophages exhibit the M1 phenotype. They release proinflammatory cytokines and mediators including TNF-α, IL-1β, IL-6, and nitric oxide (NO) to enhance bactericidal activity and phagocytosis (40). In contrast, M2-phenotype macrophages promote tissue repair by inducing TGF-β to activate fibroblasts, and inhibit inflammatory responses through secreting the anti-inflammatory cytokine IL-10 (41). To achieve intestinal homeostasis, the immune system must maintain tolerance to food-derived antigens and antigens produced by the commensal microbiota. During this process, intestinal macrophages usually present a “tolerant” phenotype, which is characterized by low responsiveness to TLR ligands (42). Furthermore, these cells continuously secrete the critical anti-inflammatory cytokine IL-10 while suppressing the production of proinflammatory cytokines and NO.

Innate lymphoid cells (ILCs) represent a highly heterogeneous subset of lymphocytes (43). As key innate immune cells residing in the intestinal lamina propria, ILCs can be classified into three core subsets (ILC1, ILC2, and ILC3) Among them, ILC3 is the most abundant ILC subset in the intestine and the major source of IL-22 secretion (44). Upon activation by microorganisms or cytokines such as IL-23, ILC3 secretes IL-22 in an antigen-independent manner. This molecule binds to the IL-22R1/IL-10R2 receptor on the surface of intestinal epithelial cells, thereby activating the STAT3 signaling pathway. Once phosphorylated, STAT3 translocates to the nucleus, where it not only upregulates the transcriptional level of the Mucin 2 (*Muc2)* gene but also promotes the post-translational processing and secretion of the Muc2 protein (45). Increased Muc2 expression significantly enhances the thickness and structural integrity of the outer mucosal layer. It not only forms a physical barrier to block the invasion of luminal bacteria and toxins into epithelial tissues and inhibit bacterial adhesion but also promotes the colonization of beneficial intestinal flora to maintain microbial homeostasis, ultimately improving the intestinal barrier’s defense capacity against pathogens.

Notably, the regulation of ILC3 function by the microbiota is partially mediated through various cell types such as epithelial cells, macrophages, and dendritic cells (46). Meanwhile, a growing body of evidence indicates that the function of ILC3 is regulated by metabolites produced by the commensal microbiota. Microbiota-derived metabolites and dietary stimulants can bind to retinoic acid receptors free fatty acid receptors, and aryl hydrocarbon receptors (AhR) to trigger ILC3. It has been clearly established that the activation of the AhR is one of the important regulatory mechanisms of ILC3 (47). For example, tryptophan metabolites such as indole-3-acetic acid can regulate ILC3 function by activating AhR (48). Recently, several research teams have reported that SCFAs can regulate the activity of ILC2 and ILC3; therefore, bacteria producing SCFAs can all regulate ILC3 function to a certain extent (49). In addition, peptidoglycan fragments released by segmented filamentous bacteria can bind to receptors on the surface of ILC3, directly stimulating the MyD88-dependent signaling pathway, thereby promoting ILC3 proliferation and the secretion of cytokines such as IL-17A and IL-22 (50).

Among all subsets of ILCs, ILC2 (type 2 innate lymphoid cells) also plays a crucial role in maintaining intestinal homeostasis. It is mainly activated by cytokines such as interleukin-33, interleukin-25 and thymic stromal lymphopoietin secreted by intestinal epithelial cells. Once activated, ILC2 secretes type 2 cytokines including IL-4, IL-5, IL-9 and IL-13 (51)—among which IL-13 can promote the proliferation of intestinal epithelial cells and enhance the tight junctions between cells, thereby strengthening the mucosal barrier (52).

As a core component of intestinal adaptive immunity, B cells regulate the intestinal microbiota through multiple pathways. In terms of microbiota selection, plasma cells—mainly derived from B2 lymphocytes —in the intestinal lamina propria secrete secretory immunoglobulin A (sIgA) (53). This sIgA can specifically bind to the surface antigens of beneficial bacteria (e.g., *Bifidobacterium* and *Lactobacillus*), interact with the polymeric immunoglobulin receptor on IECs, and anchor to mucins in the mucus layer, thereby enabling the enrichment of beneficial bacteria on the intestinal mucosa (54). Meanwhile, studies have shown that regulatory B cells (Bregs) secrete various cytokines such as IL-10, IL-35, and TGF-β to inhibit the proliferation of pathogenic T cells and immune cells including DCs and monocytes, thereby exerting an immunosuppressive effect on immunopathology (55). In terms of eliminating harmful bacteria, sIgA can bind to the virulence factors of pathogenic bacteria (e.g., pathogenic Escherichia coli and Salmonella) to exert a neutralization effect, block their adhesion to intestinal epithelial cells, and form complexes that are excreted with intestinal peristalsis (immune exclusion). In addition, B1 cells secrete natural IgM to activate the complement system or differentiate rapidly into plasma cells to secrete IgA, thus enabling the rapid clearance of pathogenic bacteria in the early stage of infection (56).

#### 2.3.2 The impact of immune dysregulation on the gut microbiota’s structure and function

Immune dysregulation refers to the disruption of the balanced state of the body’s immune system, characterized by either excessive immune activation (e.g., in autoimmune diseases) or immune hypofunction (e.g., in immunodeficiency and aging-associated immunosenescence). This dysregulation significantly impacts the compositional structure and metabolic functions of the gut microbiota through complex regulatory networks, thereby forming a vicious cycle of “immune-microbiota” crosstalk.

During excessive immune activation, the body mounts an exaggerated immune response to “self-antigens” or “harmless microbiota-derived antigens,” releasing large quantities of proinflammatory cytokines (e.g., TNF-α, IL-6, IFN-γ)—a process that directly disrupts the colonization balance of the gut microbiota. Furthermore, excessive immune activation impairs the integrity of the intestinal epithelial barrier, leading to increased intestinal permeability. This permeability not only allows opportunistic pathogens, which are normally sequestered within the intestinal lumen (such as Escherichia coli and Proteus species belonging to the Enterobacteriaceae family), to adhere to the damaged epithelial surface and proliferate extensively but also facilitates the translocation of microbial components, particularly lipopolysaccharide (LPS) (57). As a key cell wall component of Gram-negative bacteria (including Enterobacteriaceae and AIEC), LPS is a potent PAMPs that can translocate across the compromised intestinal epithelium into the systemic circulation or local submucosal tissues. Once translocated, LPS binds to TLR4 on immune cells (e.g., macrophages, dendritic cells) and intestinal epithelial cells, triggering the activation of NF-κB signaling pathway (58). This further amplifies the production of proinflammatory cytokines (e.g., TNF-α, IL-1β, IL-8) and chemokines, exacerbating local intestinal inflammation and systemic immune dysregulation. Notably, LPS translocation creates a positive feedback loop: the sustained release of proinflammatory cytokines further damages the intestinal barrier, promotes the overgrowth of Gram-negative opportunistic pathogens (increasing LPS production), and enhances LPS translocation—thus aggravating the “immune-microbiota” vicious cycle (59). Concurrently, proinflammatory cytokines alter the redox microenvironment of the intestinal tract (e.g., elevated levels of reactive oxygen species, thereby conferring a competitive advantage to oxidation-tolerant harmful bacteria. For instance, in patients with RA, the abundance of Prevotella copri in the gut is significantly elevated; its metabolic byproducts can activate Th17 cells to secrete IL-17, which further exacerbates joint inflammation (60). Similarly, in patients with inflammatory bowel disease (IBD, including Crohn,s disease and ulcerative colitis), adherent-invasive E. coli (AIEC) is enriched in the intestinal microbiota (61). This pathogen can penetrate the intestinal mucosa, triggering sustained inflammation.

## 3 Impact of gut microbiota-mediated immune regulation on PMO

**Figure 3:**
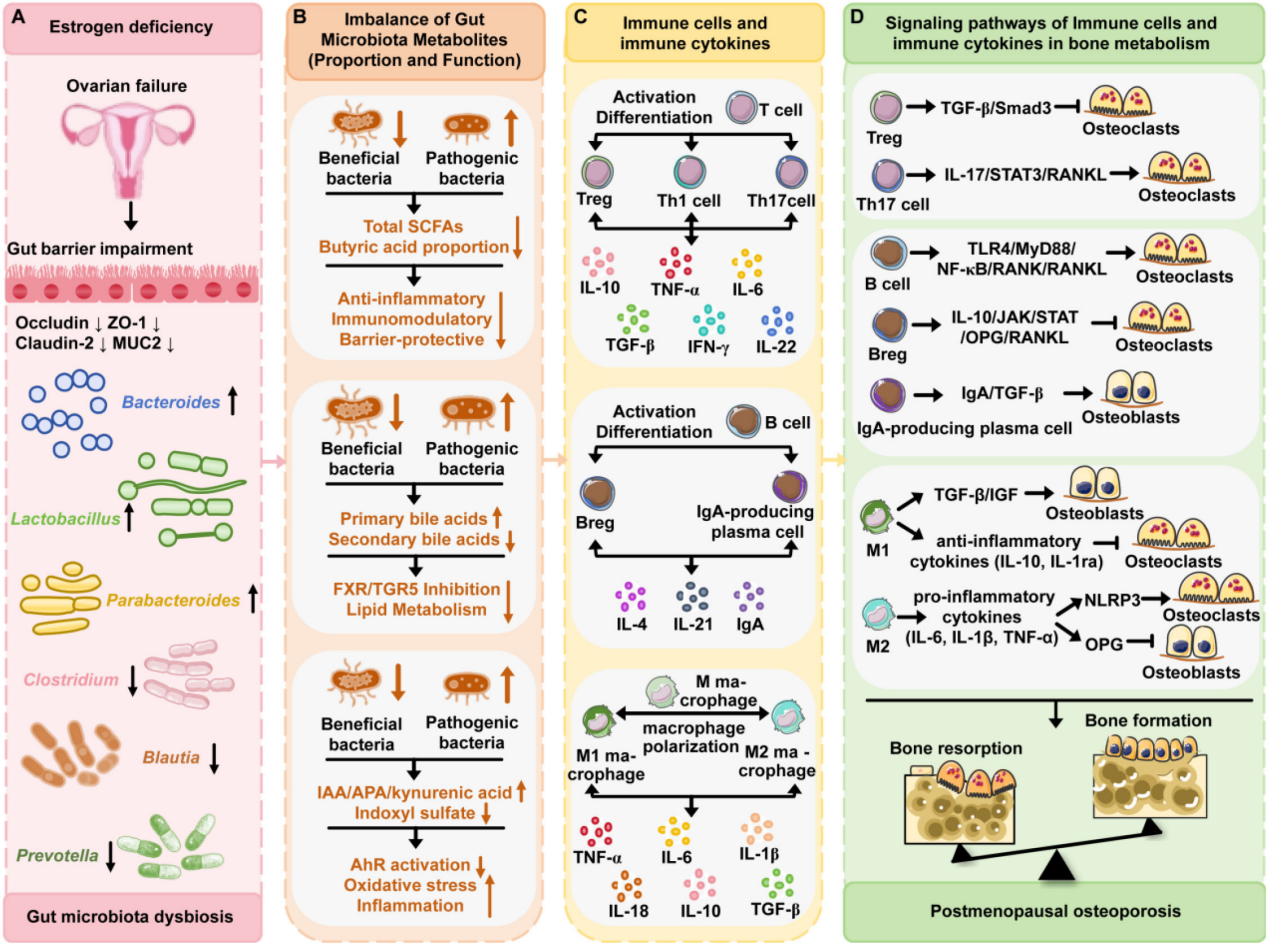
Modulations of gut microbiota, immune cells and cytokines and their effects in postmenopausal osteoporosis. **(A)** Alterations in gut microbiota during the postmenopausal period. **(B)** Dysbiosis in the composition and function of gut microbiota-derived metabolites. **(C)** Modulations of immune cells and cytokine profiles under conditions of gut microbiota dysbiosis. **(D)** The signaling pathways of immune cells and cytokines in mediating postmenopausal osteoporosis via regulating bone metabolism.

PMO is a typical pathological manifestation of bone metabolism imbalance caused by estrogen deficiency (1). Recent studies have shown that in addition to its direct effects on bone cells, estrogen deficiency reshapes the structure of intestinal flora and activates systemic immune-inflammatory responses, which constitutes an emerging paradigm for the regulation of bone homeostasis via the “Gut-Bone Axis” (**Figure 3**) (62).

### 3.1 Cascade effects of estrogen deficiency on gut microbiota and immune homeostasis

In postmenopausal women, the marked decline in estrogen levels—triggered by ovarian dysfunction—does not merely induce menopausal syndrome (e.g., hot flashes and insomnia); it also sustains a tight bidirectional regulatory interplay with intestinal microbiota dysbiosis. Jianquan He et al. conducted a study on 106 postmenopausal women (including 33 patients with osteoporosis, 42 with osteopenia, and 31 with normal BMD) by integrating 16S rRNA gene sequencing and Liquid chromatography-mass spectrometry (LC-MS)-based metabolomics analysis, and the results showed that bacterial richness and diversity in postmenopausal women with osteoporosis were decreased; additionally, there were significant differences in the abundance levels at the phylum and genus levels in the intestinal microbial community (13, 63).Specifically, *Parabacteroides*, *Lactobacillus*, and *Bacteroides* were found to be enriched in patients with PMO, whereas the abundance of Blautia, Clostridium, Lachnospiraceae_UCG-001, Lachnospiraceae_UCG-004, and Prevotella was decreased. These findings are consistent with those of another relevant study, which shows that the abundance of Prevotella in the gut microbiota of PMO women is significantly reduced, while that of Bacteroides is relatively higher (64). Furthermore, the rat OVX model has provided robust evidence to confirm the causal relationship: estrogen-deficient rats can recapitulate both the postmenopausal hormonal state in humans and the characteristic alterations in gut microbiota. Notably, these microbiota dysregulations are reversibly restored upon administration of exogenous estrogen, thereby revealing an association between estrogen levels and the homeostasis of gut microbiota (65). Mechanistically, Prevotella and Lactospira are likely dependent on estrogen to maintain their colonization capacity; in the absence of estrogen, their proliferation is inhibited, leading to a reduction in abundance (66). Additionally, estrogen enhances intestinal epithelial tight junctions (e.g., occludin and claudin expression) and reduces intestinal permeability. In contrast, estrogen deficiency impairs the intestinal barrier, increases endotoxin leakage, induces low-grade inflammation, promotes the growth of pro-inflammatory microbiota (e.g., certain Bacteroides species), and inhibits that of anti-inflammatory microbiota (e.g., Lactospira) (67).

The gut microbiota is not isolated; its structure and metabolic state directly influence immune system function. Under normal conditions, the gut microbiota maintains immune balance through “dialogue” with the intestinal mucosal immune system. When the intestinal microbiota in postmenopausal women exhibits the aforementioned reductions in diversity, imbalances in abundance, and alterations in composition, the rhythm of this “crosstalk” is completely disrupted, with consequent abnormalities in immune responses. Estrogen deficiency reduces the expression of key components of the intestinal barrier (occludin, zonula occludens-1 (ZO-1), claudin-2, and Muc2), leading to impaired intestinal barrier function and increased permeability. Concurrently, it activates proinflammatory M1 macrophages, triggering abnormal intestinal inflammatory responses, which in turn promote osteoclast differentiation and bone resorption, ultimately affecting BMD (68). Concurrently, this deficiency also weakens the inhibition of proinflammatory cytokines, leaving the body in a state of “low-grade chronic inflammation” (69). This persistent inflammatory environment further influences the differentiation trajectory of immune cells—specifically, the function of immune cells that normally promote bone formation is suppressed, while the activity of those capable of inducing osteoclast activation is enhanced, thereby laying the groundwork for the development of osteoporosis.

### 3.2 The role of immune cells and cytokines in gut microbiota-mediated bone metabolism

In the complex process of bone metabolism mediated by intestinal flora, immune cells and inflammation-related factors exert a profound impact on the function of osteocytes and the balance of bone metabolism through an elaborate regulatory network, thereby serving as a crucial link connecting intestinal microecology and bone health.

The dynamic balance of T cell subsets is pivotal for gut microbiota-mediated regulation of bone metabolism. Probiotics such as *Bifidobacterium* and *Lactobacillus acidophilus*—major producers of SCFAs—generate butyrate as a metabolite. This butyrate can activate the calcineurin pathway in naive T cells via the GPR43-cAMP signaling axis, thereby promoting the differentiation of Tregs and increasing their proportion (70, 71). *Lactobacillus rhamnosus* activates TLR2 on the surface of T cells via lipoteichoic acid, a component of its cell wall, thereby reducing the proportion of IL-17^+^ Th17 cells and increasing the proportion of CD25^+^ Foxp3^+^ Tregs to maintain Th17/Treg homeostasis (72). TGF-β secreted by Tregs can inhibit the rate of osteoclast differentiation in a Smad3-dependent manner and significantly enhance bone-forming capacity (73, 74). In contrast, when opportunistic pathogens (e.g., Escherichia coli) overproliferate, their cell wall component LPS acts as a PAMPs to bind TLR4. This interaction activates T cells through antigen presentation, which in turn stimulates innate immune cells such as monocytes-macrophages, ultimately inducing T cell differentiation and increasing the proportion of Th17 cells (75). IL-17 secreted by Th17 cells can activate the signal transducer and activator of transcription 3 signaling pathway in osteoblasts, thereby upregulating the expression of RANKL mRNA and enhancing osteoclast activity (76).

The function of B cells exhibits bidirectionality under the dynamic regulation of gut microbiota composition. *Bacteroides fragilis* induces the differentiation of Bregs and their secretion of IL-10 via its capsular polysaccharide A. This bacterium exerts a bone-protective effect by inhibiting the JAK/STAT pathway in osteoclast precursors and upregulating the expression of osteoprotegerin in osteoblasts (77–79). *Lactobacillus acidophilus*, on the other hand, activates GPR43 on B cells via the SCFAs it produces. This process not only promotes the secretion of IgA to neutralize pathogenic toxins but also stimulates B cells to release TGF-β, thereby accelerating bone mineralization (80, 81). Intervention with *Bifidobacterium longum* FSHHK13M1 can increase serum levels of 1,25-dihydroxyvitamin D (1,25-(OH)_2_D) and osteocalcin, upregulate the expression of molecules and pathways including vitamin D receptor (VDR), OPG, the Wnt10b/β-catenin pathway, and the runt-related transcription factor 2 (Runx2)/Osterix pathway, and downregulate the expression of the RANKL/RANK pathway—thereby promoting an increase in trabecular bone number and bone volume fraction (BV/TV) (82). In contrast, when opportunistic pathogens such as *Escherichia coli* undergo overproliferation, their LPS binds to TLR4 on B cells and activates the MyD88/NF-κB pathway, thereby exacerbating osteoclast-mediated bone resorption (83, 84).

The phenotypic switch in macrophage polarization serves as a crucial node in the regulation of bone metabolism by the gut microbiota. Probiotics such as *Lactobacillus acidophilus* and *Bifidobacterium longum* primarily regulate the polarization of macrophages toward the anti-inflammatory M2 phenotype (85, 86). The extracellular proteins isolated from *Lactobacillus acidophilus* enhance the microbial phagocytic capacity of macrophages via cathepsin K, reduce LPS-induced expression of RANKL, and promote the morphological transition of M2-type reparative macrophages under chronic inflammatory conditions (87). Furthermore, M2-type macrophages ultimately promote bone deposition through anti-inflammatory effects (such as secreting IL-10 and IL-1ra to inhibit proinflammatory factors) and activation of osteogenic signaling pathways (such as upregulating the expression of TGF-β and insulin-like growth factor (88, 89). A novel extracellular protein, EPS624, isolated from *Bifidobacterium longum*, elicits an intestinal tolerogenic response by stimulating TLR2 and establishes an anti-inflammatory microenvironment through the production of IL-10 and the expression of Foxp3 (90). Furthermore, Pam3CSK4, a synthetic ligand of TLR2, upregulates RANK in bone marrow cells to promote osteoclastogenesis (91). Studies by Zeng XZ et al. demonstrated that artesunate attenuates LPS-induced inflammatory osteoclastogenesis by inhibiting the expression of the TLR4/tumor necrosis factor receptor-associated factor 6 (TRAF6) signaling pathway (92). In contrast, the excessive proliferation of opportunistic pathogens such as *Escherichia coli* and *Klebsiella pneumoniae* drives the differentiation of macrophages toward the proinflammatory M1 phenotype (93, 94). M1-type macrophages secrete large amounts of IL-6, IL-1β, and TNF-α (95). These cytokines not only activate the NLRP3 inflammasome in osteoclast precursors to promote osteoclast fusion, but also inhibit osteoblast activity by downregulating the expression of OPG in osteoblasts (96).

To summarize, estrogen deficiency disrupts gut microbiota homeostasis, triggering a cascade of immune responses. These responses are characterized by Th17/Treg cell imbalance, B cell activation, altered macrophage polarization, abnormal pro-inflammatory cytokine secretion, and upregulated RANKL expression. Collectively, these factors promote osteoclast activation and exacerbate bone resorption. Specifically, gut microbiota dysbiosis induces overproliferation of opportunistic pathogens (e.g., Escherichia coli), which release LPS. LPS activates the TLR4-MyD88 pathway to promote Th17 cell differentiation (97, 98). Conversely, reduced levels of probiotics impair SCFAs production, thereby inhibiting Treg cell differentiation via the GPR43 pathway. This ultimately establishes a Th17-dominated pro-inflammatory microenvironment (99). Activated Th17 cells secrete IL-17, which—together with IL-6 (promotes Th17 differentiation and osteoclast precursor proliferation) and TNF-α (induces RANKL expression and inhibits osteoblast function)—amplifies inflammatory signaling (100). Additionally, LPS-polarized M1 macrophages further secrete IL-6 and TNF-α to enhance this effect (95). Furthermore, IL-17 activates the STAT3 pathway in osteoblasts, upregulating *RANKL* mRNA expression (76). Elevated RANKL binds to RANK on osteoclast precursors, activating NF-κB and MAPK pathways to promote osteoclast differentiation, fusion, and maturation (101). Notably, LPS from opportunistic pathogens also activates the TLR4-MyD88-NF-κB pathway in B cells, further enhancing osteoclast-mediated bone resorption (83, 84). This immune response cascade, involving multiple immune cells and factors, highlights the critical role of the gut-immune-bone axis in bone metabolism regulation. It provides a key mechanistic basis for understanding the link between gut microbiota dysbiosis and postmenopausal osteoporosis.

### 3.3 Immunomodulatory effects of gut microbiota metabolites on bone metabolism

The gut microbiota does not exist in isolation within the intestinal tract. It can generate a variety of bioactive substances with regulatory functions, including SCFAs, BAs, and tryptophan metabolites, by fermenting food components or metabolizing its own products. These metabolites are capable of crossing the intestinal barrier and exerting a crucial regulatory effect on the balance of bone metabolism through the key pathway of “gut microbial metabolites - immune regulation - bone metabolism”.

#### 3.3.1 The molecular mechanisms by which gut microbial metabolites regulate immune cells and their interacting cells

The microbiota constructs a “microbiota-metabolite-immunity” regulatory axis by metabolizing SCFAs (such as acetate, propionate, and butyrate), BAs, and tryptophan derivatives. It directly or indirectly regulates the activation, differentiation, and function of immune cells (T/B cells, macrophages, dendritic cells, etc.) and immune-interacting cells (epithelial cells, endothelial cells, fibroblasts, etc.) through mechanisms including receptor binding, epigenetic modification, signaling pathway regulation, and metabolic reprogramming, thereby maintaining immune homeostasis. For SCFAs, they can bind to GPR41/GPR43/GPR109A receptors to activate anti-inflammatory signaling pathways, inhibit HDACs to modify immune-related genes (102), and reshape metabolic phenotypes by regulating the balance between glycolysis and oxidative phosphorylation in immune cells, promoting the differentiation of Tregs and M2 polarization of macrophages, while suppressing the release of pro-inflammatory cytokines (29). After intestinal microbiota converts primary bile acids into secondary bile acids, the latter inhibit the maturation of dendritic cells and the activation of the NF-κB pathway through the farnesoid X receptor (FXR), regulate T cell differentiation via the G protein-coupled bile acid receptor 1 (TGR5), and enhance the expression of tight junctions in intestinal epithelial cells to reduce abnormal immune activation caused by microbiota translocation (103). Tryptophan is metabolized by the microbiota into products such as kynurenine and indoleacetic acid (IAA), which induce the secretion of anti-inflammatory cytokines and regulate the balance between Tregs and Th17 cells through the AhR, or inhibit the proliferation of effector T cells by competitively depleting tryptophan (104). In addition, these metabolites synergize with intestinal epithelial cells to secrete defensins and mucins for enhancing barrier function, interact with endothelial cells to reduce immune cell recruitment, and regulate the secretion of chemokines by fibroblasts to affect immune cell migration, forming a multicellular synergistic immune regulatory network.

#### 3.3.2 The regulatory effect of gut microbial metabolites on bone metabolism mediated by immune regulation

SCFAs, the major metabolites of dietary fiber fermented by the gut microbiota, and exert significant regulatory effects on immune cells and osteocytes (105).On the one hand, SCFAs can inhibit the release of proinflammatory cytokines (e.g., IL-6, IL-1β), and TNF-α by activating G protein-coupled receptors (GPCRs), thereby alleviating the chronic inflammatory response mediated by immune cells (106, 107). Chronic inflammation is a key driver of PMO; excessive proinflammatory factors stimulate the differentiation and activity of osteoclasts, thereby accelerating bone resorption. Therefore, the anti-inflammatory effects of SCFAs can indirectly reduce bone loss. Our previous studies also found that SCFAs can regulate the differentiation of B cells through the free fatty acid receptor 2, thereby alleviating the occurrence of skeletal inflammatory responses (108). On the other hand, SCFAs can also act directly on osteocytes, promote the proliferation and differentiation of osteoblasts, and enhance bone-forming capacity (105). Studies have shown that SCFAs can regulate the FoxO3/Wnt/β-catenin signaling pathway to provide a favorable environment for osteoblast growth, thereby maintaining the balance of bone metabolism (109). Researchers demonstrated in *in vitro* cell experiments that the supplementation of butyrate into the culture medium containing human amniotic mesenchymal stem cells (hAMSCs) significantly upregulated intracellular Runx2 expression, while notably increasing the synthesis of osteogenic-related proteins such as osteocalcin (OC) and osteopontin (OPN) (110). In animal experiments, supplementation with Bifidobacterium animalis enhanced the abundance of butyrate-producing bacteria in mice intestines and restored butyrate levels in the intestinal tract and bone tissue, and micro-computed tomography and bone histomorphometric analysis confirmed that butyrate significantly increased mice’s bone mineral density (BMD) and bone formation rate (BFR) (111). In addition, another experiment in mice fed a high-fiber diet revealed that propionate (C3) and butyrate (C4) may induce osteoclast metabolic reprogramming, which enhances glycolysis at the cost of oxidative phosphorylation, thereby downregulating essential osteoclast-specific genes (e.g., TRAF6 and NFATc1), increasing bone mass, and preventing postmenopausal bone loss (112).

BAs are cholesterol-derived molecules synthesized in the liver as primary BAs, including cholic acid, chenodeoxycholic acid, and their conjugated forms; they are further transformed into secondary BAs (e.g., deoxycholic acid, lithocholic acid (LCA), and their derivatives) under the action of the gut microbiota (113). BAs and their metabolites are involved in regulating the differentiation and function of innate and adaptive immune cells, such as macrophages (Macs), DCs, Tregs, Th17 cells, CD4^+^ Th1/Th2 cells, Bregs, and natural killer T cells (114). Dysregulation of BAs and their metabolites can drive the development and progression of various inflammation-associated diseases. Silvia Ruiz-Gaspà and her colleagues have demonstrated that bilirubin and LCA exert detrimental effects on osteoblasts by reducing the viability, differentiation, and mineralization of osteoblasts, increasing their apoptosis, and altering their gene expression (115). Mechanistically, bilirubin downregulates the expression of the *RUNX2* gene and upregulates the expression of the *RANKL* gene in bone tissue, whereas LCA only upregulates the expression of the *RANKL* gene in bone tissue. Notably, ursodeoxycholic acid is capable of neutralizing these detrimental effects induced by bilirubin and LCA. Another study demonstrated that SCFAs (e.g., acetate, propionate, butyrate) and BAs metabolites produced by Akkermansia muciniphila (A. muciniphila) promote Runx2-mediated osteoblast differentiation and inhibit NF-κB-driven osteoclastogenesis (116). Furthermore, studies have shown that the secondary bile acid 3β-hydroxydeoxycholic acid can bind to the FXR on the surface of DCs, thereby inhibiting their antigen-presenting function and reducing the differentiation of Th17 cells as well as the secretion of IL-17 (117).

Tryptophan, an essential amino acid in humans, exerts effects on immune homeostasis and bone metabolic balance through its metabolic pathways that generate distinct bioactive metabolites (118). Among these metabolic pathways, the kynurenine pathway is a critical branch of tryptophan metabolism. Its metabolites, such as kynurenine and quinolinic acid, can modulate the functions of immune cells—specifically by inhibiting the proliferation and activity of T cells and reducing the production of proinflammatory cytokines—thereby alleviating inflammatory damage to bone tissue (119). As demonstrated by Jeon C et al., elevated kynurenine levels enhance osteoclast differentiation-associated mineralization in ankylosing spondylitis and reduce RANKL-mediated osteoclast differentiation via inducing the expression of OPG (120). Furthermore, another key metabolic pathway of tryptophan is the indole pathway, through which tryptophan is metabolized by the gut microbiota (e.g., *Bifidobacterium* spp., *Clostridium* spp.) into indole and indole derivatives (e.g., indole-3-propionic acid (IPA), IAA). These substances, especially IAA, activate intestinal AhR, which effectively repairs intestinal barrier function by stimulating the Wnt/β-catenin signaling pathway. Meanwhile, supplementation with IAA and IPA can enhance M2 macrophages to secrete large amounts of IL-10, which spreads from the intestinal lamina propria to the bone marrow, thereby promoting osteoblast formation and inhibiting osteoclast formation (121). Mechanistic studies by Chen Y et al. showed that IPA improves intestinal barrier function by increasing transepithelial electrical resistance and upregulating tight junction proteins (ZO-1, claudin-1, occludin). Additionally, IPA inhibits the release of proinflammatory cytokines (IL-1β, IL-6, TNF-α) in a dose-dependent manner via regulating the TLR4/MyD88/NF-κB and TLR4/TRIF/NF-κB pathways (122, 123).

### 3.4 The role of intestinal barrier function and intestinal leakage in PMO

The intestinal barrier serves as a critical defense against the invasion of harmful substances in the intestine, comprising four primary components: the mechanical barrier, the chemical barrier, the immunological barrier, and the biological barrier (10). Among these components, the biological barrier is composed of the gut microbiota. It maintains intestinal microecological balance through mechanisms such as nutrient competition and inhibition of pathogenic bacterial colonization, while also promoting the repair of intestinal epithelium.

After menopause, estrogen levels in women decline sharply, leading to reduced diversity, altered abundance, and compositional shifts in the gut microbiota; these changes directly induce a series of impairments to the intestinal barrier. In terms of pathogenic bacterial inhibition, the reduced number of beneficial bacteria significantly impairs their ability to suppress pathogens by competing for nutrients and adhesion sites. Meanwhile, the synthesis of antimicrobial substances (e.g., bacteriocins and organic acids) secreted by *Bifidobacterium* spp. and lactic acid bacteria decreases drastically, failing to effectively inhibit the activity of harmful bacteria and thereby significantly increasing the risk of intestinal infection (124, 125). In terms of the auxiliary function of immune regulation, the interaction between the imbalanced gut microbiota and intestinal immune cells (e.g., T cells, B cells, and macrophages) becomes disrupted, failing to normally promote the maturation and differentiation of immune cells, which leads to a reduction in the release of anti-inflammatory factors (e.g., IL-10, IL-1ra) and a weakened inhibitory effect on proinflammatory factors (e.g., TNF-α, IL-1β, IL-6)—this not only impairs the defensive capacity of the intestinal immune barrier but also may induce local chronic inflammatory responses in the intestine, further damaging the intestinal mucosa (126). In terms of nutrient metabolism and substance synthesis functions, the reduction in beneficial bacteria weakens their ability to decompose substances such as dietary fiber and polysaccharides, resulting in insufficient production of SCFAs—which not only serve as the primary energy source for intestinal epithelial cells but also regulate intestinal pH, and their deficiency further impairs the proliferation and repair capacity of IECs while exacerbating the imbalance of the intestinal microenvironment (127–129). Furthermore, postmenopausal women may experience significant emotional fluctuations due to hormonal changes, and these negative emotions can impair intestinal neuromodulation through the gut-brain axis, disrupt the stability of the intestinal microecology, and impair function of the intestinal biological barrier (130).

When intestinal barrier function is impaired and “leaky gut” occurs, harmful substances in the intestine, particularly gut microbiota-derived endotoxins (mainly LPS), undergo translocation into the bloodstream (131). As a core component of the cell wall of Gram-negative bacteria, endotoxin exhibits a potent immune-activating effect; once it enters the systemic circulation, it is rapidly recognized by TLR4 on the surface of immune cells, triggering a cascade reaction of downstream inflammatory signaling pathways and thereby prompting immune cells such as macrophages and monocytes to release large quantities of inflammatory factors (132). The abnormal elevation of proinflammatory factors disrupts bone metabolism homeostasis: TNF-α and IL-1 can directly stimulate the differentiation and activation of osteoclasts, enhancing their bone resorption function; IL-6, on the other hand, indirectly accelerates osteoclast formation by promoting the proliferation of osteoclast precursor cells, while inhibiting the activity of osteoblasts and reducing bone matrix synthesis (133–135). Furthermore, LPS partially promotes RANKL-induced osteoclast differentiation through the upregulation of C-X-C chemokine receptor type 4 (CXCR4) (136). Therefore, endotoxin translocation induced by leaky gut and the subsequent inflammatory response constitute one of the key pathological mechanisms driving the development and progression of PMO.

## 4 Bidirectional regulation between osteogenic factors and gut microbiota

There is a close bidirectional regulatory network between bone and gut microbiota, which is specifically reflected in the shaping effect of bone-derived factors on the homeostasis of gut microbiota, as well as the reverse regulatory effect of gut microbiota and their metabolites on the secretion of bone metabolism-related factors.

A recent study found that the serum level of osteocalcin is correlated with the Chao index of gut microbiota in patients with Crohn’s disease (137), further indicating that osteocalcin may affect the composition of microbiota and optimize the structure of gut microbial community. As a core hormone for bone metabolism, active vitamin D can directly target intestinal epithelial cells and immune cells. By enhancing the integrity of intestinal barrier and inhibiting excessive inflammatory response, it creates a suitable microenvironment for the colonization of beneficial bacteria, thereby maintaining the homeostasis of gut microbiota (138).

Gut microbiota can reversely regulate the secretion of bone-derived cytokines and hormones through their metabolites and participation in hormone activation processes. On the one hand, SCFAs produced by the fermentation of dietary fiber by beneficial intestinal bacteria can act on bone through the circulatory system. They can not only promote osteoblasts to secrete osteocalcin, but also inhibit the expression of cytokines related to osteoclast activity (such as receptor activator of NF-κB ligand, RANKL), thereby regulating the balance of bone metabolism (139).

On the other hand, gut microbiota can participate in the activation process of vitamin D. The latest study found that Bifidobacterium adolescentis CCFM1447 can increase the level of VD metabolites in fermentation supernatant, converting 25-hydroxyvitamin D synthesized by the liver into biologically active 1,25-dihydroxyvitamin D. The activated vitamin D can further reversely regulate the activity of osteoblasts and osteoclasts, indirectly affect the secretion of bone-derived cytokines, and form a complete regulatory loop (140).

## 5 Gut microbiota-based intervention strategies and their mechanisms of action

### 5.1 Probiotic intervention

Probiotics, primarily encompassing *Lactobacillus* (e.g., *L. acidophilus*, *L. rhamnosus*), *Bifidobacterium* (e.g., *B. longum, B. bifidum*), and other genera such as *Streptococcus thermophilus*, *Bacillus subtilis*, and *Saccharomyces boulardii*, exert multidimensional effects on host physiology through intricate mechanisms (**Table 1**).

**Table 1:** The role of different intervention strategies in bone metabolism in postmenopausal osteoporosis.

Intervention measures	Representative strains/Components	Research models	Implementation methods	Bone metabolism	References
Probiotics	*Lactobacillus* species: *Lactobacillus acidophilus*, *Lactobacillus rhamnosus*; *Bifidobacterium* species: *Bifidobacterium longum*, *Bifidobacterium bifidum*; other genera: *Streptococcus thermophilus*, *Bacillus subtilis*	Ovariectomized (OVX) Sprague-Dawley (SD) rats; OVX C57BL/6 mice	*Butyricicoccus pullicaecorum* (BP) (4.55×10^9^ CFU/kg) was gavaged orally for 12 weeks; *Bacillus coagulans* suspension (10^9^ CFU) was gavaged continuously for 60 days.	BP improved femoral stereological parameters in OVX rats, including significantly increased femoral mass and volume, elevated total osteocytes and osteoblasts, and reduced osteoclasts; BC improved the 3D microstructure of the proximal femoral metaphysis in OVX mice, with significant increases in whole-body bone mineral density (BMD), trabecular bone volume fraction (BV/TV), and trabecular thickness (Tb.Th).	(149, 150)
Prebiotics	Inulin, Fructooligosaccharides (FOS), Galactooligosaccharides (GOS), Xylooligosaccharides (XOS), Lactulose	Female C57BL/6 mice; SD rats	Fed a diet supplemented with 10% (w/w) FOS; Fed diets containing 0, 2, 4, 6, or 8% GOS for 8 consecutive weeks.	Diet supplemented with FOS significantly increased BMD, tibial length, and BV/TV, as well as serum levels of bone turnover markers procollagen type I N-terminal propeptide (P1NP) and C-telopeptide of type I collagen (CTX-1); Diet supplemented with GOS significantly improved net absorption and retention rates of calcium and magnesium, and enhanced distal femoral BMD.	(164, 166)
Fecal Microbiota Transplantation (FMT)	The intact microbial community in feces from healthy donors (including bacteria, fungi, viruses, etc.)	OVX C57BL/6 mice; Germ-free female C57BL/6 mice	Recipient mice were gavaged daily with 200 μl of bacterial suspension (10^8^ CFU/ml, from feces of healthy donor mice) or 200 μl of diluted supernatant (from postmenopausal women with normal bone mass or osteoporosis) for 8 consecutive weeks.	BMD, BV, and BV/TV were increased. The distal femoral medullary cavity was reduced, with increased trabecular bone and elevated cortical thickness (Ct.Th), indicating alleviated bone loss. Decreased osteoclast differentiation and increased osteoblast number suggested enhanced bone formation.	(168, 169)
Traditional Chinese Medicine (TCM)	Single Herbs: Epimedium Herb, Quercetin, Eucommia Bark; Classic Prescriptions: Shengu Granule, Jiangu Granule, Xianlinggubao Capsule	OVX SD rats	Shengu granule (12 g/kg·d) was administered orally for 14 consecutive weeks; Jiangu granule extract was gavaged for 6 or 12 consecutive weeks; Xianlinggubao capsule was administered continuously at 1 g/kg·d for 3 months.	The number and morphology of trabecular bone were significantly improved, along with increased BMD, tissue mineral density (TMD), BV, and BV/TV.	(170–172)
Moxibustion	Moxa Wool	OVX C57BL/6 mice; OVX SD rats	Conducted by professional personnel, gentle moxibustion and other techniques were applied to acupoints including Guanyuan (CV4) and Sanyinjiao (SP6).	Moxibustion enhances the activity of bone marrow mesenchymal stem cells (BMSCs), increases the level of bone glaprotein (BGP), and restores bone metabolism balance.	(173, 175)

#### 5.1.1 The role of probiotics in regulating gut microbiota and immunity

In terms of modulating intestinal microecology, probiotics contribute to the expansion of beneficial bacterial populations in the gut by two primary means: first, they promote the growth of endogenous favorable microbial communities and directly increase the abundance of beneficial bacteria via their own proliferation (141). For instance, intestinal *Lactobacillus* stimulates lactic acid production, which activates hypoxia-inducible factor (HIF)-2α-mediated signaling pathways to improve intestinal health (142). Second, they mediate intestinal homeostasis through competitive exclusion—a natural phenomenon involving the competition for nutrients and ecological niches—which enhances the colonization of beneficial bacteria while suppressing the growth of pathogenic microorganisms (143). Beyond this, they also inhibit pathogenic colonization by producing antimicrobial metabolites, such as SCFAs, reuterin, and bacteriocins (144, 145). In the context of immunomodulation, probiotics regulate both the innate and adaptive immunity of the host. For innate immunity, they activate PRRs (e.g., TLRs) on immune cells to modulate the secretion of cytokines (e.g., IL-6, IL-10) (146). For adaptive immunity, they balance the Th1/Th2 cell subsets, promote the differentiation of Treg cells, and increase the production of sIgA (147).

#### 5.1.2 Probiotics regulation immune prevention and treatment PMO clinical trials and animal experimental evidence

Recent studies have indicated that probiotics, particularly species of *Lactobacillus* and *Bifidobacterium*, show potential for alleviating PMO through immunomodulatory mechanisms and the gut-bone axis. Per-Anders Jansson and colleagues confirmed that in a 12-month intervention study, early postmenopausal women received a Lactobacillus strain or placebo once daily. The results showed that compared with the placebo group, treatment with the Lactobacillus strain significantly reduced the loss of lumbar spine bone mineral density (LS-BMD), with a mean difference of 0.71% (95% confidence interval [CI]: 0.06–1.35) (148). Notably, animal experiments using the OVX model have demonstrated that the combination of Coprococcus (a gut commensal bacterium associated with probiotic effects) and 3-hydroxyanthranilic acid effectively inhibits osteoclastogenesis, maintains bone mass, and prevents the development of PMO. This effect is mediated by regulating the gut microbiota and balancing Th17/Treg cell populations, which in turn reduces the levels of proinflammatory cytokines (IL-6, TNF-α) while increasing the production of anti-inflammatory factors (IL-10, TGF-β) (149). Leena Sapra et al. found in their study that supplementation with the probiotic *Bacillus coagulans* can significantly improve BMD, bone strength, and bone microstructure by regulating the anti-osteoclastogenic potential, immunosuppressive capacity, and immunomodulatory properties of Bregs. Additionally, it inhibits inflammatory bone loss in OVX mice through enhancing the effects of SCFAs. Furthermore, via modulation of the “gut-immune-skeleton” axis, *Bacillus coagulans* effectively alleviates inflammatory bone loss even under conditions of PMO (150). Meta-analysis revealed that interventions with *Lactobacillus* and *Bifidobacterium* significantly increased BMD and BV/TV in ovariectomized animals. Although the effect of these probiotics did not reach statistical significance, they showed a trend toward promoting bone formation and inhibiting bone resorption. Furthermore, *Lactobacillus* exhibited a more significant effect size (151).

### 5.2 Prebiotic intervention

2016, the International Scientific Association for Probiotics and Prebiotics defined dietary prebiotics as substrates that are selectively utilized by host microorganisms to confer health benefits (152). This category encompasses a diverse range of compounds, which are primarily classified based on their chemical structures and sources, with their mechanisms of action focusing on the selective modulation of host-associated microorganisms. The primary classes of dietary prebiotics encompass fructooligosaccharides (FOS) (153), galactooligosaccharides (GOS) (154), and xylooligosaccharides (XOS) (155), inulin (156), lactulose (157), resistant starch (158), polyphenols (159), and glucooligosaccharides (160).

#### 5.2.1 The role of prebiotics in regulating gut microbiota and immunity

As functional food components resistant to degradation by digestive enzymes in the human upper gastrointestinal tract, prebiotics exert regulatory effects on gut microbiota through the synergy of multi-dimensional mechanisms. Regarding substrate supply: they can be preferentially taken up by specific beneficial gut bacteria (e.g., *Bifidobacterium*, *Lactobacillus*, *Faecalibacterium* spp.) and serve as carbon and energy sources, thereby directionally promoting the proliferation and activity enhancement of these bacterial groups (161). For instance, specific prebiotics (e.g., non-digestible arabinoxylan oligosaccharides) regulate the metabolic pathways of gut microbiota to facilitate the biosynthesis of functional metabolites (e.g., spermidine). *In vitro* and *in vivo* studies have confirmed that spermidine can regulate the expression of genes associated with autophagy, immunity, and inflammation in intestinal epithelial cells, thereby promoting the colonization of beneficial gut microbiota (162). In terms of intestinal barrier function, prebiotics upregulate the expression of tight junction proteins between intestinal epithelial cells, thereby strengthening mucosal barrier integrity and reducing the translocation of toxins and pathogenic bacteria (163). At the level of immune regulation, prebiotics can promote the proliferation of beneficial bacteria like *Bifidobacterium* and *Lactobacillus*. These bacteria produce SCFAs through fermentation. These SCFAs have been shown to enhance the activity of Bregs, which is crucial for maintaining immune tolerance and suppressing excessive inflammatory responses. Additionally, prebiotics can promote the activation and proliferation of immune cells (e.g., macrophages, DCs, and Tregs) in the lamina propria of the intestinal mucosa, while regulating the balance between anti-inflammatory and proinflammatory cytokines.

#### 5.2.2 Research progress on prebiotics improving bone health via the gut microbiota

The protective effect of FOS against osteoporosis has been validated in multiple animal studies. The latest research findings indicate that dietary supplementation with 10% FOS for 8 consecutive weeks can increase the systemic bone mineral content and trabecular bone in the proximal tibial metaphysis and lumbar vertebrae (164). Tanabe et al. observed that FOS treatment alleviated high-fat diet-induced bone loss, reversed the imbalance in the differentiation of osteoblasts, adipocytes, and osteoclasts, and improved intestinal barrier function by reducing the downregulation of tight junction proteins and the increase in inflammatory factors (165). As a naturally occurring prebiotic in human milk, GOS differ from FOS, which mainly serves as a fermentable substrate. GOS exerts multi-dimensional regulatory effects on mineral homeostasis through a complex interaction network with gut microbiota, making it an ideal candidate for enhancing mineral absorption and retention rates in different populations. Animal experimental data further confirmed the calcium and magnesium retention effects of GOS: After Sprague-Dawley rats were fed a diet containing GOS, net magnesium absorption, femoral bone ^45^Ca uptake, and retention of calcium and magnesium were significantly increased. Consistently, the total volumetric bone mineral density (vBMD) of the distal femur, cancellous vBMD and area, as well as the vBMD of the proximal tibia were all significantly increased with dietary GOS supplementation (166). 2’-Fucosyllactose ameliorates aging-related osteoporosis by restoring the diversity of gut microbiota, increasing the abundance of *Bifidobacterium, Prevotellaceae*, and *Akkermansia*, inhibiting the growth of Stenotrophomonas, and suppressing the secretion of proinflammatory factors (167).

### 5.3 Fecal microbiota transplantation

FMT is a therapeutic strategy that involves transferring functional gut microbiota from healthy donors to recipients, aiming to restore microbial homeostasis in dysbiosis-associated diseases. Studies by Yuan-Wei Zhang et al. have shown that FMT treatment can reshape the state of gut microbiota and effectively ameliorate bone loss in OVX-induced osteoporosis mice. The underlying mechanisms may involve correcting gut microbiota dysbiosis, increasing fecal levels of SCFAs, optimizing intestinal permeability, and inhibiting the release of osteoclastogenic cytokines, thereby suppressing excessive osteoclast formation (168). In another study, Tinglong Chen et al. transplanted gut microbiota from PMO patients into sham-operated mice and found that the recipient mice exhibited increased intestinal permeability, impaired intestinal mucosa, and reduced expression levels of tight junction proteins ZO-1 and claudin in the intestinal barrier. Their findings indicated that the gut microbiota from PMO patients accelerates bone mass loss in mice (169). Preclinical studies using OVX rodent models have demonstrated that FMT from healthy donors can reverse OVX-induced reductions in beneficial bacteria (*Lactobacillus*, *Bifidobacterium*, *Faecalibacterium prausnitzii*) and increases in proinflammatory bacteria (*Escherichia coli*, *Desulfovibrio*). It also restores fecal SCFAs—particularly butyrate—and inhibits osteoclastogenesis by downregulating RANKL and reducing proinflammatory cytokines (TNF-α, IL-6). Additionally, FMT can rebalance immune dysregulation in OVX models, promote Tregs differentiation (via SCFA-mediated upregulation of Foxp3), and inhibit Th17 polarization, thereby reducing osteoclast activity (168).

Overall, FMT holds promise as a microbiota-targeted therapy for PMO, and larger-scale randomized trials are warranted to validate its long-term efficacy and its ability to reduce fracture risk.

### 5.4 Other intervention measures

#### 5.4.1 Traditional Chinese medicine

In recent years, as research on the association between gut microbiota and bone health has deepened, the role and mechanism of TCM compound prescriptions in improving osteoporosis have attracted increasing attention in the academic community. Multiple studies have revealed that these prescriptions exert their effects by regulating gut microbiota, immune cell differentiation, and related metabolites, as well as through other signaling pathways. Studies by Xiao Cong Chen et al. demonstrated that after administration of Shengu Granules, the diversity of gut microbial communities in osteoporotic rats was enhanced, while the abundance of intestinal proinflammatory bacteria was reduced. The potential mechanism involves that Shengu Granules upregulate the expression of FOXP3 (which regulates Treg cell differentiation) and increase the levels of SCFAs (which modulate Th17 cell differentiation), thereby ameliorating the impact of the Th17/Treg axis on osteoporosis (170). Additionally, Pan Sun et al. found that Jiangu Granules, by modulating gut microbiota homeostasis and enriching SCFA-producing probiotics, reduce intestinal epithelial permeability, restore the Treg/Th17 cell ratio, and inhibit osteoclast differentiation, ultimately achieving the effect of preventing and treating PMO (171). Xian-Ling-Gu-Bao capsule (XLGB) has been confirmed to regulate lipid and bile acid metabolism, thereby providing a scientific basis for the treatment of osteoporosis (172).

#### 5.4.2 Acupuncture

In recent years, moxibustion-mediated regulation of gut microbiota has emerged as a research hotspot in the field of TCM. Studies have shown that moxibustion may affect bone metabolism by regulating the structure and composition of gut microbiota, thereby improving bone pathological conditions. Animal experiments have confirmed that moxibustion can enhance the osteogenic differentiation capacity of bone marrow mesenchymal stem cells (BMSCs) in ovariectomized female rats, increase the level of bone glaprotein (BGP), and thus elevate bone mass through the Wnt/β-catenin signaling pathway (173). With the deepening understanding of the “gut-bone axis”, the role of gut microbiota and their important metabolites in the occurrence and progression of PMO has attracted increasing attention (174). The latest study has discovered and confirmed that TCM moxibustion therapy may inhibit postmenopausal bone loss by regulating the level of gut microbiota-derived serotonin, activating the 5-Hydroxytryptamine 2A receptor, and promoting the osteogenic differentiation of BMSCs (175).

TCM intervention approaches have improved PMO by regulating gut microbiota; however, they still face multiple challenges, including batch-to-batch variations in herbal components, lack of standardization in acupuncture protocols, and limited understanding of strain-specific mechanisms. Future efforts should focus on strengthening research on active components, conducting large-scale clinical trials, and exploring their synergistic effects with other therapies.

## 6 Challenges and prospects

### 6.1 Current challenges in research

Gut microbiota-mediated immune regulation has provided a novel perspective for research on PMO, yet its translational progress from basic theory to clinical application still faces multiple critical challenges. The limitations of current research mainly lie in three dimensions: insufficient depth of mechanistic elucidation, weak clinical evidence, and technical barriers in translational application.

At the mechanistic level, the precise molecular network governing microbiota-immune-osteometabolism remains incompletely clarified. Although the roles of Th17/Treg cell balance dysregulation and impaired intestinal barrier function in PMO pathogenesis have been confirmed, the specific mechanisms by which certain microbial members selectively regulate immune cell differentiation via metabolites remain largely unclear. For instance, the association between abnormal abundance of *Streptococcus* and the degree of bone loss in PMO patients has been observed, but the causal chain by which it affects osteoclast differentiation signaling pathways through specific metabolic intermediates (e.g., SCFAs, tryptophan metabolites) or virulence factors (e.g., capsular polysaccharides) remains undefined.

The weakness of clinical translational evidence significantly hinders the implementation of intervention strategies. Existing probiotic intervention studies generally suffer from small sample sizes and short intervention durations, leading to insufficient statistical power for BMD improvement effects and poor result reproducibility. An analysis of a study on the intestinal Bifidobacterium communities of subjects from six Asian regions revealed inherent differences in the intestinal flora composition among subjects from different regions, with their intestinal Bifidobacterium communities exhibiting regional specificity in response to probiotics—some probiotic strains exert differential regulatory effects on the intestinal Bifidobacterium communities of populations in different regions (176). A more prominent issue is the interindividual heterogeneity in microbiota regulation—gut microbiota composition is shaped by multiple factors such as genetic background, dietary patterns, and lifestyle, resulting in a 30%-70% variation in response rates to probiotic interventions among PMO patients, which severely impedes the establishment of standardized treatment protocols.

### 6.2 Future research directions and prospects

Based on current research foundations and bottlenecks, future breakthroughs should focus on three directions, driven by multidisciplinary integration. The core of mechanistic research is to decipher the precise signaling axes among microbial metabolites, immune cells, and osteocytes. Clinical translational research needs to establish an integrated multi-omics analysis system—through correlation analysis of metagenomics, metabolomics, immunomics data with large-scale clinical cohorts, to clarify the causal network of specific microbial markers, key metabolic nodes, and immune regulatory pathways associated with bone loss in PMO patients, thereby identifying core intervenable targets (177).

In terms of technological innovation, the development of novel microbiota-regulating tools is crucial to overcoming existing intervention bottlenecks. Nanodelivery systems (e.g., polyethylene glycol-modified nanoemulsions, PNEs) exhibit excellent intestinal targeting and microbiota-regulating efficacy, but their biocompatibility optimization and large-scale production processes still require breakthroughs (178). Additionally, CRISPR-Cas9-based precise microbiota editing technology offers the possibility of specifically modifying metabolic functions of key microbiota, holding promise for precise regulation of bone metabolism (179). He Bin et al. isolated BMSCs from osteoporotic rats, cultured them separately, extracted exosomes from these cells for miRNA analysis, and subsequently identified miR-151-3p and miR-23b-3p as potential key regulators of bone metabolism. This indicates its potential in treating postmenopausal osteoporosis by targeting and regulating miR-151-3p and miR-23b-3p in BMSC-derived exosomes (180). Furthermore, bioengineered probiotics can be developed via gene-editing technologies to enable efficient expression of bone-protective bioactive molecules, thereby directly or indirectly regulating the gut-bone axis signaling.

As the core metabolites of dietary fiber fermented by gut microbiota, SCFAs exert bone-protective effects through multiple pathways, such as regulating intestinal barrier integrity, inhibiting systemic chronic inflammation, and acting directly on osteoblasts/osteoclasts. However, natural SCFAs have inherent limitations, including high water solubility, rapid absorption in the gastrointestinal tract, short half-life (only 1–2 hours), and rapid metabolic inactivation by the liver, which severely restrict their clinical application. To address these issues, SCFAs analogs can be developed via chemical structure modification or dosage form optimization. These analogs retain the bone-protective activity of natural SCFAs while significantly improving bioavailability and targeting, making them promising therapeutic agents with greater clinical potential.

Gut microbiota-mediated immune regulation provides a novel intervention dimension for PMO prevention and treatment. Advances in this field will profoundly reshape the clinical management model of bone metabolic diseases, with significant scientific and social significance. With the deepening of mechanistic research and breakthroughs in translational technologies, this field is expected to achieve leapfrog development from theoretical innovation to clinical application in the future.

## 7 Conclusions

A drastic decline in estrogen levels following menopause serves as the primary initiating factor of PMO. Meanwhile, gut microbiota, acting as a pivotal mediator, exerts a profound impact on bone metabolic homeostasis by reshaping the immune microenvironment. Currently, PMO intervention strategies targeting gut microbiota have demonstrated considerable potential, and existing basic research has further validated their feasibility. However, significant translational bottlenecks remain unresolved. Moving forward, leveraging multidisciplinary integration and technological innovations is expected to facilitate the establishment of a novel “gut microbiota-targeted intervention” system for PMO prevention and treatment. This system will thereby offer safer and more effective clinical strategies to improve bone health in postmenopausal women.

## Glossary

PMO: Postmenopausal osteoporosis
RA: rheumatoid arthritis
SCFAs: Short-chain fatty acids
HDACs: histone deacetylases
DCs: dendritic cells
GALT: gut-associated lymphoid tissue
Tregs: regulatory T cells
Th17: T helper 17
PRRs: pattern recognition receptors
PAMPs: pathogen-associated molecular patterns
DAMPs: damage-associated molecular patterns
TLRs: toll-like receptors
MyD88: myeloid differentiation primary response 88
NF-κB: nuclear factor-κB
MAPKs: mitogen-activated protein kinases
TNF-α: tumor necrosis factor-α
IL-10: interleukin
IL-6: interleukin-6
IL-1β: interleukin-1β
MD-2: myeloid differentiation factor 2
LPS: lipopolysaccharide
TRIF: TIR-domain-containing adapter-inducing interferon
TRAM: TRIF-related adapter molecule
TBK1: TANK-binding kinase 1
IKKϵ: inhibitor of nuclear factor-κB kinase ϵ
IRF3: interferon regulatory factor 3
IL-18: interleukin-18
Th1: T helper 1
NLRP3: NOD-like receptor family pyrin domain containing 3
NLRs: nucleotide-binding oligomerization domain-like receptors
TRAF6: tumor necrosis factor receptor-associated factor 6;
ASC: apoptosis-associated speck-like protein containing a CARD
NOD2: Nucleotide-binding oligomerization domain 2
TGF-β: Transforming growth factor-β
RegIIIγ: Regenerating islet-derived protein III gamma
IFN-γ: interferon-γ
NO: nitric oxide
CD40: cluster of differentiation 40
ILCs: Innate Lymphoid Cells
IECs: Intestinal Epithelial Cells
IgA: Immunoglobulin A
sIgA: secretory immunoglobulin A
Bregs: Regulatory B cells
LC-MS: Liquid Chromatography-Mass Spectrometry
OVX: Ovariectomized
Foxp3: Forkhead Box P3
GPR43: G Protein-Coupled Receptor 43
BAs: Bile Acids
LCA: Lithocholic Acid
FXR: Farnesoid X Receptor
IAA: Indole-3-Acetic Acid
AhR: Aryl Hydrocarbon Receptor
IPA: Indole-3-Propionic Acid
RUNX2: Runt-Related Transcription Factor 2
BMD: Bone Mineral Density
BV/TV: Bone Volume Fraction
FOS: Fructooligosaccharides
GOS: Galactooligosaccharides
XOS: Xylo-Oligosaccharides
HMOs: Human Milk Oligosaccharides
vBMD: Volumetric Bone Mineral Density
FMT: Fecal Microbiota Transplantation
TCM: Traditional Chinese Medicine
BMSCs: Bone Marrow Mesenchymal Stem Cells
BGP: Bone Glaprotein
EPS: exopolysaccharides
PSA: Polysaccharide A
*Muc2*: Mucin 2.
